# Can Flowering Greencover Crops Promote Biological Control in German Vineyards?

**DOI:** 10.3390/insects8040121

**Published:** 2017-11-03

**Authors:** Christoph Hoffmann, Janine Köckerling, Sandra Biancu, Thomas Gramm, Gertraud Michl, Martin H. Entling

**Affiliations:** 1 Julius Kühn-Institute, Institute for Plant Protection in Fruit Crops and Viticulture, Geilweilerhof, D-76833 Siebeldingen, Germany; janine.koeckerling@julius-kuehn.de (J.K.); sandra.biancu@julius-kuehn.de (S.B.); thomas.gramm@julius-kuehn.de (T.G.); gertraud.michl@julius-kuehn.de (G.M.); 2 Institute for Environmental Sciences, University of Koblenz-Landau, Fortstr. 7, D-76829 Landau, Germany; entling@uni-landau.de

**Keywords:** habitat management, European grapevine moth, egg-predator, *Trichogramma*, predatory mite, *Typhlodromus pyri*, *Itoplectis alternans*, *Lobesia botrana*

## Abstract

Greencover crops are widely recommended to provide predators and parasitoids with floral resources for improved pest control. We studied parasitism and predation of European grapevine moth (*Lobesia botrana*) eggs and pupae as well as predatory mite abundances in an experimental vineyard with either one or two sowings of greencover crops compared to spontaneous vegetation. The co-occurrence between greencover flowering time and parasitoid activity differed greatly between the two study years. Parasitism was much higher when flowering and parasitoid activity coincided. While egg predation was enhanced by greencover crops, there were no significant benefits of greencover crops on parasitism of *L. botrana* eggs or pupae. Predatory mites did not show an as strong increase on grapevines in greencover crop plots as egg predation. Overall, our study demonstrates only limited pest control benefits of greencover crops. Given the strong within- and between year variation in natural enemy activity, studies across multiple years will be necessary to adequately describe the role of greencover crops for pest management and to identify the main predators of *L. botrana* eggs.

## 1. Introduction

Highly simplified agroecosystems often lack vital resources for beneficial organisms such as predatory arthropods, with negative consequences for pest regulation. In turn, the activity of functionally important arthropods can be enhanced through the integration of floral resources into farming systems [1,2,3]. However, flowering plants may also enhance pest species and compete for water with crops, challenging their viability as a pest control option in viticulture [4].

Parasitoids and predators can play an important role in the regulation of vineyard pests. Adult parasitoids may be limited within the landscape or the vineyard by the availability of adequate food resources [5] or alternative hosts that enable natural enemies to switch between the target pest and other non pest-species [6]. Silvestri [7] already showed in 1912 that planting cabbage within the vineyard rows enhanced the parasitism rate of European grapevine moth (*Lobesia botrana*) in vineyards by providing an alternative host species for the pupal parasitoid *Dibrachys affinis* (Hymenoptera: Pteromalidae) as this species has more generations per year than *L. botrana* and therefore needs alternative host species to survive within the vineyard.

**Figure 1:**
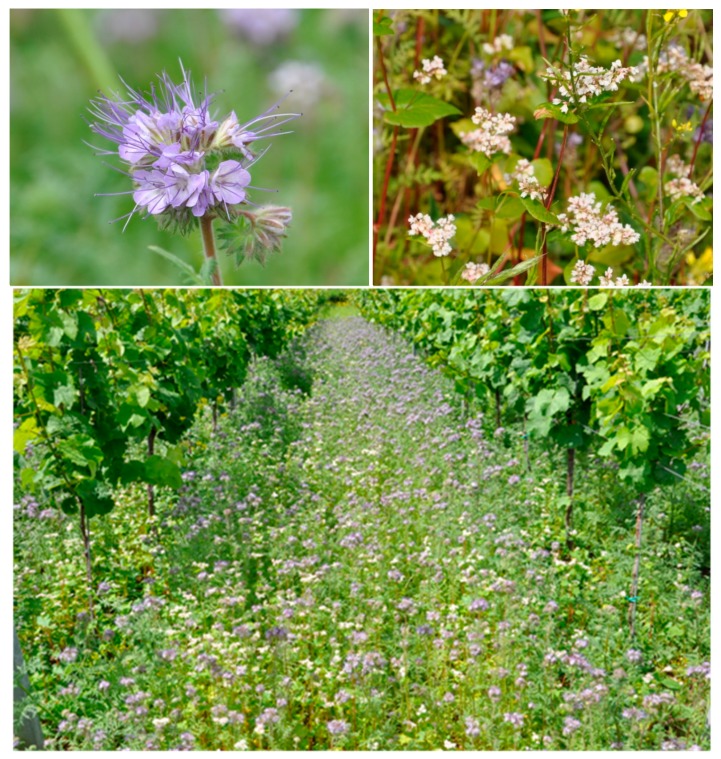
The greencover crops *Phacelia tanacetifolia* and *Fagopyrum esculetum* (8 weeks after sowing, location: Siebeldingen, Germany).

We tested whether beneficial insects (parasitoid wasps and predators) and predatory mites can be promoted within vineyards by planting greencover crops *Fagopyrum esculentum* (Caryophyllales: Polygonaceae) and *Phacelia tanacetifolia* (Asterales: Boraginaceae) (Figure 1). These two plant species are commonly used as greencover in German organic viticulture [8]. Predatory mites play a crucial role in the regulation of pest mites (Tetranychidae and Eriophyidae) and are known to be enhanced by greencover and pollen availability in vineyards [9]. Even though they are not directly involved in the regulation of *L. botrana*, they might still be an indicator for further beneficial effects of green cover management. To our knowledge, the predicted beneficial effect of greencover crops on the biological control of *L. botrana* has not yet been experimentally tested. The idea behind planting greencover crops is that they supply parasitoids and predatory mites with additional food resources (nectar, pollen and alternative hosts), thereby enhancing their performance against crop pests [6]. *Phacelia tanacetifolia* is known to promote parasitoid performance in potato fields [10] and is one of the most studied plant species in habitat management trials [11]. In addition to their positive effects on natural enemies, greencover crops may directly reduce pest infestation through their interference with host finding by the pest (resource concentration hypothesis [12]).

Previous studies of greencover crops for conservation biological control of lepidopterous pests in vineyards have yielded mixed results. It was shown, for example, that buckwheat (*F. esculentum*) planted within the rows of vineyards increases the abundance of the parasitoids that control certain endemic leafroller species (Lepidoptera: Tortricidae) in New Zealand [13]. Nevertheless, the rate of parasitism of the target pest (other leafroller species) did not change between plots with and without *F. esculentum*. This suggests that natural enemy activity is not necessarily linked between greencover and the grape canopy, or it suggests that no relationship is observable at the scale at which the study was conducted.

Within our trial we focused mainly on *Lobesia botrana* as a pest. As we wanted to stay focused in this paper on our target pest we did not cite all the abundant papers (e.g., review in [11]) in which *F. esculentum* and *P. tanacetifolia* were used to enhance beneficial effects against other target pests. 

Relevant natural enemies of *L. botrana* include parasitoid wasps (here mainly *Itoplectis alternans* (Hymenoptera: Pimplinae) and *Trichogramma* spp. (Hymenoptera: Trichogammatidae)), predatory insects (Neuroptera: Chysopidae, Hymenoptera: Formicidae, Heteroptera: Anthocoridae) and predatory mites (mainly *Typhlodromus pyri* (Acarina: Phytoseiidae)). In addition to these groups, we included other leaf-inhabiting fauna because of their potential role as alternative food for natural enemies. For predatory mites, pollen that is blown from the greencover onto vine leaves can play an important role as an additional food source [14]. Our aim was to test if there is a measurable ecosystem service of the two greencover plants (sown once or twice a season) compared to the spontaneous vegetation of our vineyards. If so, it could be a useful tool for integrated pest control in central European vineyards to use these plants as greencover. The first step was to identify the naturally occurring species of natural enemies. The next step was to see whether flower bloom coincided with the key activity periods of natural enemies. A further step was to check if greencover management affects densities of natural enemies. Last step was to evaluate the effect of greencover crops on arthropod densities of the grapevine itself. We tested the following hypotheses:

(1) Densities of pest control agents such as Phytoseiid mites in the canopy, and the predation and parasitism of eggs and larvae of *Lobesia botrana* on the grapes are enhanced by flower-rich greencover crops.
(2) Densities of potential pests such as *Epitrimerus vitis*, *Eriophyes vitis* and Thysanoptera are reduced by greencover crops.

These experiments were part of a European project (PURE: Pesticide Use-and risk Reduction in European faming systems with integrated Pest Management; www.pure-ipm.de) to evaluate innovations for integrated viticulture [15]. The idea of this project was to conduct a scientific feasibility and efficacy test for greencover crops as an IPM tool to enhance biological control of pests. As such, the failure of germination after drilling is considered as a result but not as a failure of the trial as well as the sometimes missing co-occurrence of flowering greencover crops with the presence of natural enemies. 

## 2. Materials and Methods 

### 2.1. Experimental Design

**Figure 2:**
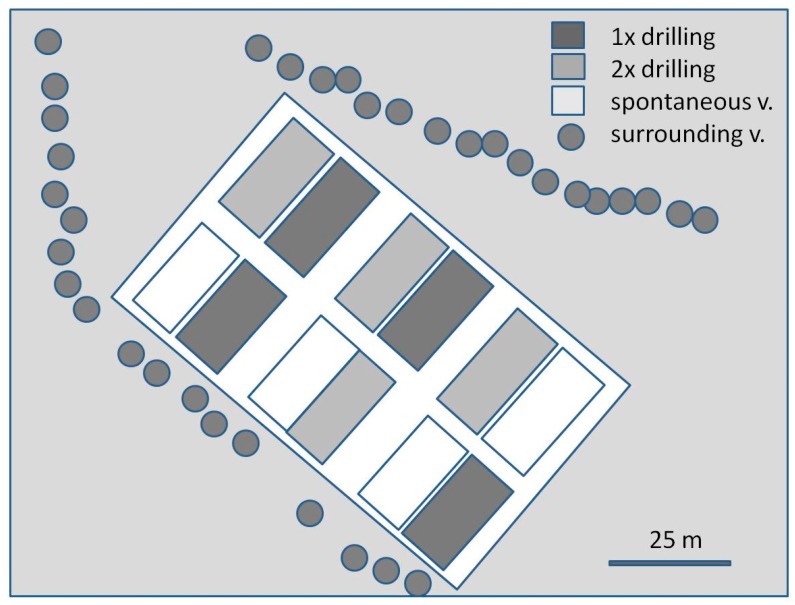
Design of the greencover field experiment. Four replicates each were established from (i) one and (ii) two drillings of *Phacelia tanacetifolia* and *Fagopyrum esculentum* per season, compared to a control of (iii) spontaneously growing ground vegetation. The white space between the plots (width of 10 m) was vegetated with grass that was kept short by mowing.

In 2013 and 2014 we established four replicates of three treatments in an experimental vineyard (variety: Riesling, planted 2010) in Siebeldingen, Germany. We chose this vineyard because it is equipped with an irrigation system, which we used to avoid water competition between greencover crops and the vines. That way, it was not necessary to mow the greencover for water supply reasons. The treatments included (i) one and (ii) two drillings of *Phacelia tanacetifolia* and *Fagopyrum esculentum* per season, compared to a control of (iii) spontaneously growing ground vegetation. The spontaneous vegetation consisted mainly of Poaceae and typical vineyard weeds like *Solanum nigrum*, *Amaranthus*, *Epilobium*, *Rumex*, *Taraxacum*. Each plot consisted of 7 rows of grapes with one additional separation-row between two plots. The interrow distance was two meters. Each row had a length of 25 m and included 25 grape vines. The treatments were applied randomly but under the condition that directly adjoining plots have different treatments, and that each of the three possible treatment pairs occurs twice (Figure 2). Sampling was done exclusively in the centre of the two innermost rows of each plot. Prior to the planting of greencover crops, all plots were tilled in the first week of April of 2013 and 2014. *Fagopyrum esculentum* (50 kg seed/ha) and *Phacelia tanacetifolia* (15 kg seed/ha) were sown in alternating rows in the greencover crop treatments, using seeds prepared from Becker & Schöll (Ilsfeld, Germany). The two cover crops were broadcast sown by hand. To obtain an even coverage, the seeds were portioned in plastic bags for each row. After sowing, the soil was reworked by a circular harrow to a depth of around 5 cm. Both cover crop treatments were sown once in April (24 April 2013 and 13 April 2014), and the two-sowings treatment was sown again in July–August (24 July 2013 and 8 August 2014). Figure 2 illustrates the arrangement of the plots (see Figure S1 for more details). The sampling took place in the central part of each plot to avoid edge effects. The distance between two sampling sites within one plot was 4 m. To get an idea whether there was a species loss from the surrounding area into the vineyard, we also sampled the surrounding trees and shrubs using the same methods as in the plots.

The plant protection regime was uniform across all plots. Conventional fungicides were chosen with an emphasis on protecting phytoseiid mites (Table S1). For the control of *L. botrana,* we used the mating disruption product RAK 1 + 2 (BASF). Herbicides (2× glyphosate) were applied to a 30 cm strip directly underneath the grapevines. The greencover crops grew on around 85% of the ground surface.

### 2.2. Timing of Flowering, Parasitoid Activity and Presence of L. botrana

Flowering periods of the greencover crops were documented each year through weekly surveys (2013; 2014: calendarweek 13–44). Parasitism of *L. botrana* was measured in the experimental plots as well as in the surrounding area within hedgerows, grassland and trees and compared to the presence of *L. botrana* instars by using a BASF pheromone trap that was located 1 km outside the trial plot (Lure with E7/Z9 dodecenyl acetate). To estimate the possible presence of larval instars, we considered the beginning and the end of the male flight. We used the first catches of males within these traps as a starting point for a phenological model [16] that allowed us to estimate the development of the instars of *Lobesia* in the field. As we used pheromones in the trial plot to control *L. botrana* (see Section 2.1), the natural population of this species was on a very low level and we did not check the relative abundance of naturally occurring *L. botrana*. 

### 2.3. Rearing of L. botrana and Measuring Field Activity of Pupa-, Egg-Parasitoids and Egg Predators

**Figure 3:**
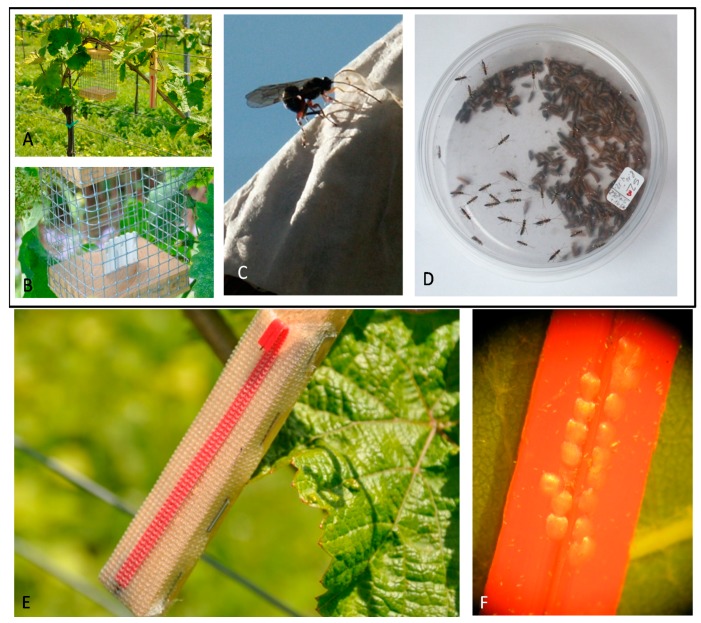
Measuring the activity of pupal and egg parasitoids of the European grapevine moth *Lobesia botrana*. (**A**,**B**) Wire cages to avoid bird predation of *L. botrana* pupae; (**C**) Pupal parasitoid of *L. botrana* (*Itoplectis alternans*) during oviposition on corrugated paper containing pupae; (**D**) Pupal parasitoids emerging in the lab; (**E**) *L. botrana* egg strips concealed under gauze to measure egg parasitism; (**F**) Exposed *L. botrana* egg strips to measure egg predation.

The laboratory rearing of *L. botrana* was accomplished according to Markheiser et al. [17]. Corrugated paper was used as pupation substrate for the larvae [18] and eggs were deposited on polyethylene sticks [19]. The eggs exposed were not older than one day. The age of the pupae was between one and four days at the moment of exposure. Eggs and pupae were then exposed to naturally occurring parasitoids and predators in the experimental vineyard plots. In order to avoid predation by birds, corrugated paper with *L. botrana* pupae was enclosed in small cages constructed of coarse mesh. Five cages (mesh size: 10 mm) per plot and week were supplied with distinct numbers of pupae (Figure 3) so that the pupae were accessible. After one week in the field, exposed pupae were brought back to the laboratory and reared in *Drosophila*-breeding tubes until *L. botrana* and/or parasitoids emerged. Parasitism was determined by dividing the number of emerged parasitoids by the number of exposed pupae. Eggs of *L. botrana* were similarly enclosed in fine polyester mesh gauze (0.8 mm mesh size) in order to avoid predation during exposure to parasitoids in the vineyard. To evaluate egg predation, another set of *L. botrana* eggs was simultaneously placed in the vineyard without any gauze covering. Therefore, a total of 10 polyethylene sticks with and 10 without gauze covering (20 sticks total) were placed in each experimental plot for a one-week period (240 per week in total). The egg predation rate was then quantified by comparing egg counts on the sticks before and after exposure in the vineyard. The same was done in the surrounding trees (*Alnus glutinosa, Prunus domestica*) and shrubs (*Rubus fruticosus, Hippophae rhamnoides, Prunus domestica subsp. syriaca*) which were not treated with plant protection products (*n* = 20 sites per week). The distance between the sampled shrubs and trees of the surrounding vegetation to the vineyard is between 10 m and 60 m. In both years, egg and pupae cards were placed in the experimental plots between calendar week 13 and 44. The mean number of exposed instars was 31 ± 14 eggs/stick and 3.4 ± 1.2 pupae/cage. In the two years 18,772 pupae and 100,084 eggs were exposed for one week within the trial. For the measurement of the predation rate, we compared the number of eggs before and after exposure (desiccated—, not desiccated—and parasitized eggs are left). In most cases we could find destroyed empty eggs where only the basal part of the egg was left. If larvae emerge from the eggs, the whole egg shell is left on the stick [20]. In one week of June of 2014, larvae had already emerged from the eggs. Thus, measurements from this week represent parasitism but not predation rates. In the remaining weeks we counted damaged as well as absent eggs as “predated eggs”. The pupal parasitoid was determined to family and genus level (using keys from [21,22,23,24]); for the species of Pimplinae, we used the key of [25]. 

*Trichogramma* species that emerged from parasitized sentinel eggs were determined using molecular methods following references [26,27]. The DNA of individual wasps was extracted with the Chelex method. The ITS 2 region of the internal rDNA was amplified using the primers Tricho for TGTGAACTGCAGGACACATG and Tricho rev GTCTTGCCTGCTCTGAG established by [28]. For a 25 μL PCR amplification, we used Dynazyme II DNA Polymerase (Thermo Fisher scientific, Waltham, MA, USA), applying the conditions stated in the user guidelines with primers at 0.5 M concentration and 50 °C annealing temperature in 30 cycles. RFLP analysis of the PCR product was performed by restriction digestion with FastDigest Mnl I (Thermo Fisher scientific, Waltham, MA, USA) and with MseI (Thermo Fisher scientific, Waltham, MA, USA) in reactions containing 13 μL of the PCR product, 1× buffer and 10 units of enzyme, incubated for 25 min at 37 °C and followed by 5 min 95 °C denaturation. PCR and restriction products were electrophoresed in 2% agarose gel visualized with SERVA DNA stain clear G. In one case, when MnlI cut differed from [26], we sequenced the PCR product and determined the species with the NCBI Database. The taxonomy of *Trichogramma* spp. follows Fauna Europaea [29].

### 2.4. Leaf Mesofauna

To sample the leaf mesofauna, 25 leaves per month and plot were sampled in 2014 (June–October) randomly in the middle of the canopy. The samples (*n* = 4 per treatment) were taken in the morning from the middle of an inner row (e.g., row 4 of 7) of each plot by taking the leaves from 12 to 15 different plants (see Figure 2B). Leaves were transferred into water with detergent and washed according to the method of [30]. All arthropods were then identified at the family level and Eriophyid mites to the species level with a stereomicroscope (Zeiss Stemi 2000, Carl-Zeiss GmbH, Oberkochen, Germany). For species determination of Phytoseiidae and Tydeidae, random samples of adult predatory mites were separated and stored in 70% ethanol. These adult mites were then cleared with lactic acid and mounted on slides using Hoyers Fixative for embedding [31]. The mites were identified using a fluorescent light microscope (Leica DM 4000 B, Leica Microsystems, Wetzlar, Germany) following the key provided by [32]. The total number of mite individuals determined to the family level was 26,968. The number of Eriophyid mites determined to species level was 796, of Tydeidae 162 and of Typhlodromidae 762, meaning that 6.4% of the total mites were determined to the species level. As the species composition did not differ between the variants, we chose not to display these results in detail.

### 2.5. Data Analyses

All analyses were carried out in R for Mac OS version 3.2.1 [33]. Differences in arthropod density and in the leaves, egg- and pupal parasitism between greencover crop treatments and the control were tested with one-way ANOVA. To avoid non-independence among repeated measures and subsamples within each of the *n* = 12 treatment plots, we summed numbers of individuals and averaged parasitism and predation rates for each plot and over time. Accumulating the data from multiple samples into one value for each plot avoids problems of non-independence of the repeated measures in the same plot. To test if the spatial pairing of the plots affected our analysis, we also performed mixed-effect models with pairs 1–6 as a random factor. As the results were similar, we decided to present only the simple one-way ANOVA results. Pairwise differences between treatments were determined with Tukey’s HSD tests. When there was a clear temporal separation of occurrence within years, we analyzed the respective time periods separately. Given the failure of the second sowing of the greencover crop in 2014 (see Section 3.1.2), the treatments with one and two sowings were combined for that year. However, we kept the two greencover crop treatments separate due to the possibility that arthropod (e.g., mite) populations from 2013 year affected densities and predation pressure in 2014, and because drilling of the greencover crop in August 2014 could affect arthropods on the grapevines via changes such as altered microclimate. Examination of Q-Q plots and residuals versus factor levels revealed that no data transformation was necessary. Results were regarded significant at *p*-values < 0.05.

## 3. Results

### 3.1. Co-Occurrence of Beneficial Insects, Pest Stages and Flowering of Greencover Crops

#### 3.1.1. Year 2013

**Figure 4:**
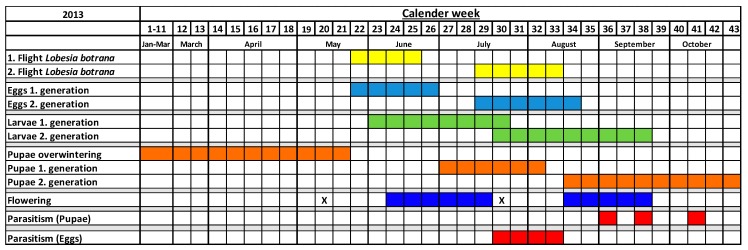
Phenology of the different *Lobesia botrana* developmental stages (yellow, light-blue, green, orange), egg- and pupal parasitoids (red), drilling (= X) and flowering periods of the greencover crops (blue) (*Phacelia*, *Fagopyrum*) in 2013.

Figure 4 shows that only for the second drilling in July 2013 was there is a coincidence of parasitoid activity and flowering of the greencover crops. The activity of *Trichogramma* egg-parasitoids overlapped with the naturally occurring *Lobesia botrana* eggs for four weeks but did not overlap with the flowering time of the greencover crops. *L. botrana* pupal parasitoids were also active outside the flowering of greencover crops in 2013; their activity began in September, when the last flowers of the second drilling had already wilted. 

#### 3.1.2. Year 2014

**Figure 5:**
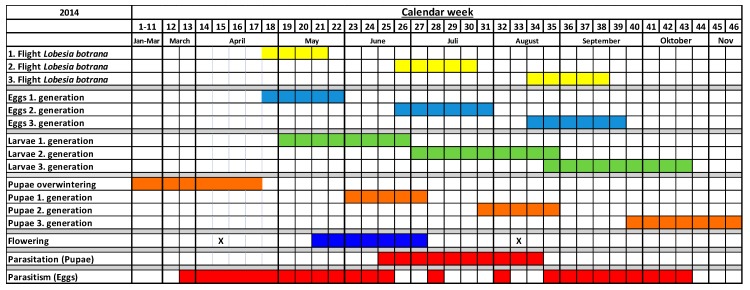
Phenology of the different *Lobesia botrana* developmental stages (yellow, light-blue, green, orange), egg- and pupal parasitoids (red), drilling (= X) and flowering periods of the greencover crops (blue) (*Phacelia*, *Fagopyrum*) in 2014.

In 2014 the second drilling of the greencover crops failed to develop due to dry weather conditions (Figure 5). Unlike 2013, a year with colder spring temperatures, in 2014 there was a co-occurrence of parasitoid activity and flowering of the greencover crops between May and July. The activity of *Trichogramma* egg-parasitoids overlapped during 11 weeks with the naturally occurring *Lobesia botrana* eggs and during 5 weeks with the flowering period of *Fagopyrum* and *Phacelia*. The same observation was made for the pupal parasitoids. Their activity in 2014 began earlier in June and overlapped during three weeks with the flowering of the greencover crops. 

### 3.2. Egg-Parasitoids

**Figure 6:**
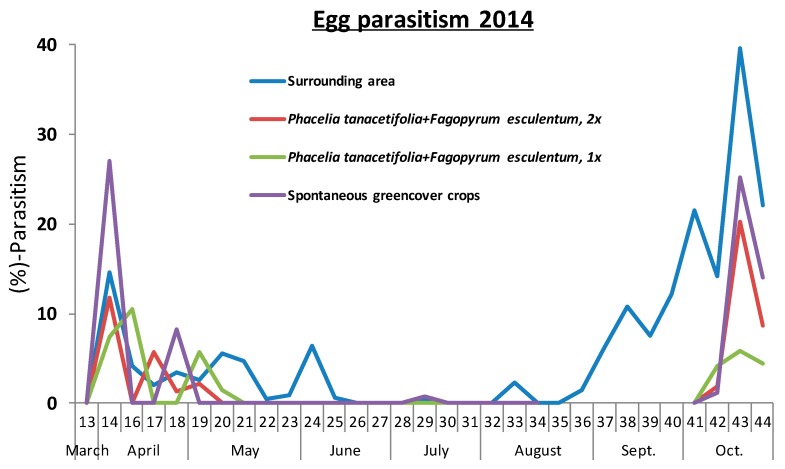
Phenology of *Trichogramma* sp. reared from *L. botrana* eggs in the different cover crop treatments during 2014.

Parasitism of *L. botrana* eggs in 2013 was negligible, with only two events of parasitism in the first and second week of September, respectively, not exceeding 0.5% parasitism rate. In contrast, the egg parasitism rate in 2014 reached 40% (Figure 6). We found four species of *L. botrana* egg parasitoids, with a strong dominance of two species within the vineyard (Table 1).

**Figure 7:**
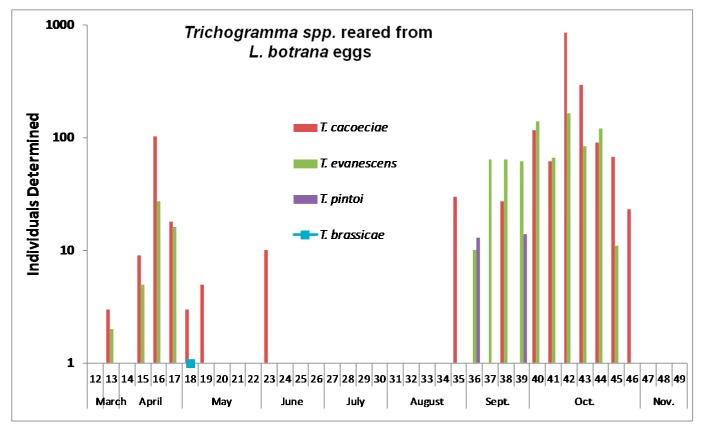
Phenology of *Trichogramma* spp. reared from *L. botrana* eggs during 2014.

The activity of the two dominant *Trichogramma* species peaked in April and October 2014, respectively, while the less abundant *T. pintoi* was largely restricted to autumn (Figure 7). *T. pintoi* was not found within the vineyard but in the surroundings (Table 1). Overall parasitism did not differ significantly between treatments in the early (calendar weeks 13–21: F_2,9_ = 0.76, *p* = 0.498) or in the late season (calendar weeks 41–44: F_2,9_ = 0.36, *p* = 0.708).

### 3.3. Pupal Parasitoids

**Figure 8:**
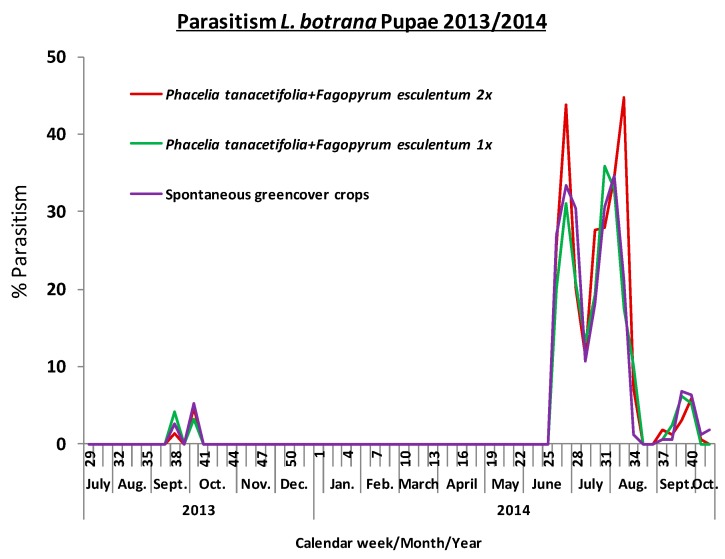
Phenology of pupal parasitism of *L. botrana* within the greencover crop treatments.

Parasitism of *L. botrana* pupae was also substantially lower in 2013 than in 2014 (Figure 8). It never exceeded 5% in 2013, but increased to 45% in 2014. In both years, parasitism was restricted to the time between June and October. All parasitism was caused by *Itoplectis alternans* (Hymenoptera: Ichneumonidae). There was no measurable benefit of the greencover crops for the parasitism of *L. botrana* pupae (F_2,9_ = 0.10, *p* = 0.903; numbers from 2013 were too low for analysis).

### 3.4. Predators and Other Arthropods

**Figure 9:**
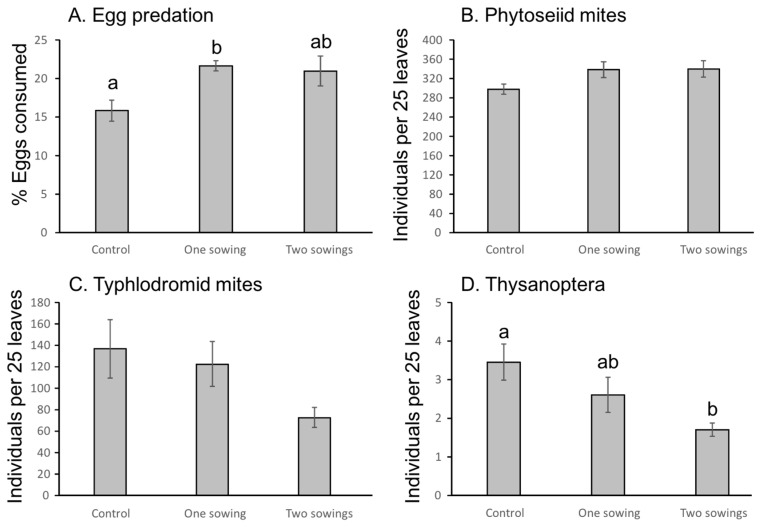
Effects of greencover crops on (**A**) *Lobesia botrana* egg predation and on numbers of (**B**) Phytoseiid mites, (**C**) Tydeid mites and (**D**) thrips (Thysanoptera) in 2014. Means ± 1 standard error.

Predation of *L. botrana* eggs was significantly enhanced by greencover crops compared to spontaneous vegetation (F_2,9_ = 4.88, *p* = 0.0367, Figure 9A). Phytoseiid mites tended to have higher densities in the greencover crop treatments (Figure 9B), but the difference was not significant (F_2,9_ = 2.51, *p* = 0.136). Tydeidae tended to have lower numbers under two sowings of greencover crops (Figure 9C), but this was again not significant (F_2,9_ = 2.68, *p* = 0.122). No effects of greencover crop treatments were found for *Epitrimerus vitis* (F_2,9_ = 1.28, *p* = 0.324) or *Eriophyes vitis* (F_2,9_ = 1.75, *p* = 0.227). Densities of Thysanoptera decreased with increasing number of greencover sowings (Figure 9D, F_2,9_ = 5.08, *p* = 0.0334).

**Figure 10:**
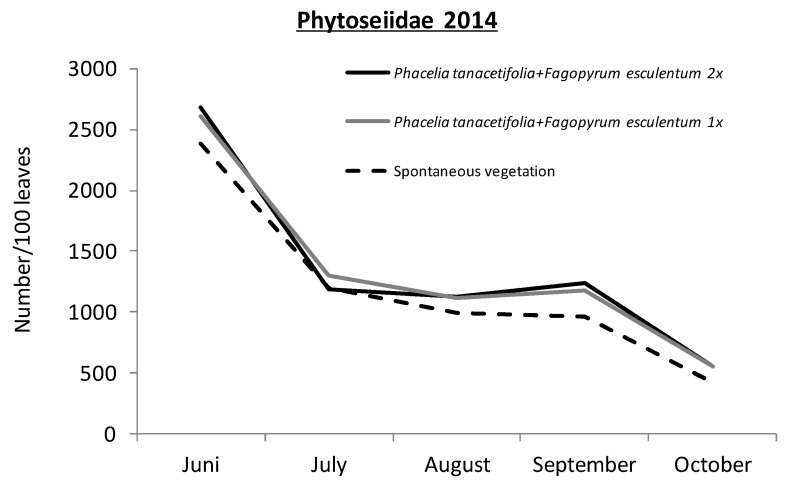
Density of phytoseiid mites (mainly *Typhlodromus pyri*) on grapevine leaves of the different greencover crop treatments.

The abundance of predatory mites in the greencover crop trials was exceptionally high. Up to 25 mites per leaf were counted in June 2014 and at least 10 mites per leaf in September 2014 (Figure 10). More than 95% of the randomly sampled predatory mites were *Typhlodromus pyri* (Table S2). All the other organisms found within the leaf mesofauna are listed in Table S3.

## 4. Discussion

Natural enemies in vineyards can be limited by the availability of floral resources, prey, or host organisms [6]. At a population level, the phenological synchronization of the three trophic levels parasitoid—host insect—host plant can be a limiting factor for survival of a parasitoid species within the ecosystem. Our results show that the parasitic wasps *Trichogramma* and *Itoplectis* are partly active during the flowering of *Phacelia* and *Fagopyrum* greencover in viticulture, with strong differences between the two sampled years. While the greencover crops flowered two times in 2013 when sown twice, in 2014 the second drilling was not successful. Nevertheless, in 2013 there was only low activity of parasitoids during the flowering phase of the greencover crops. This contrasted with high parasitoid activity before, during and after the flowering of the greencover crops in 2014. The years differed in weather conditions in a way that *L. botrana* developed only two generations in 2013, but three in 2014 (see Figure 4 and Figure 5). In 2013—according to our model estimation—*L. botrana* eggs occurred in late May whereas in 2014 adults hatched already in late April. One reason for the different rates and periods of parasitism between these years could be a lack of synchronization of the phenology of the parasitoids with *L. botrana* in 2013. In the year 2014, egg parasitism occurred independently from the flowering of the greencover crops. As uncovered soil is not a viable alternative in the trial region in Germany due to trafficability of the rows, we did not check this treatment in comparison with the greencover crops. If our results could be confirmed elsewhere it would mean that spontaneously growing greencover crops might not be worse than expensive sowings.

We think that the lack of egg parasitism during the summer months of 2014 was not a side effect of fungicide sprayings because the activity of the parasitoids was the same in the non-treated surroundings as in the intensively treated vineyard.

Predatory mites are, phylogenetically, soil mites [32]. On woody plants like trees, shrubs or grapes, they are often limited by plant protection products, food and humidity [32]. As plant protection was identical across our vineyard, this factor is not further considered here. It has already been shown that greencover crops can enhance the mite density on grapes compared to bare soil [13]. The reason for this might be that greencover crops cool down the soil by evaporation (own unpublished data) and thus allow the mites to settle on the lower trunk of the grapevine without desiccation. Another reason might be that flowering plants deliver pollen which is a food source for predatory mites in the absence of animal prey [34]. Although mite densities did not differ between treatments, the mite population was extremely high compared to untreated wild *Vitis* spp. in the US [35]. This may be due to the abundant spontaneously growing coverplants even in the plots where no greencover crop was sown. The spontaneously growing ground vegetation consisted mainly of Poaceae and typical vineyard weeds like *Solanum nigrum*, *Amaranthus*, *Epilobium*, *Rumex*, *Taraxacum;* all produce pollen that can adhere to the hairy underside of the grape leaves [36].

Zhang et al. [37] showed that the longevitiy of *Trichogramma* spp. was increased while feeding on nectar and pollen. Begum et al. [5] showed that the longevity of *Trichogramma carverae* increased when the adults feed on flowers of *F. esculentum* and *Lobularia maritima*. In release experiments, parasitism rates of *T. carverae* on eggs of *Epiphyas postvittana* were significantly higher in plots with than in plots without flowers. In literature on organic viticulture, it is often argued that flowering greencover crops within vineyards can enhance beneficial insects like parasitic Hymenoptera in the greencover crops [8]. In our trial, the diversity of the *Trichogramma* species parasitizing *L. botrana* eggs and that of the pupal parasitoids did not change significantly between the different greencover variants. We could not measure any positive effects on the parasitism of *Lobesia* eggs or pupae on the grape plant. The rate of parasitism of *L. botrana* eggs and pupae by parasitoids during the season and over the two years (2013–2014) fluctuated immensely. Parasitism peaks also occur without flowering greencover crops. 

As flight distance of insects increases with body size [38], we expect *I. alternans* (body length > 10 mm) to fly over much longer distances than *Trichogramma* spp. (body length < 1 mm). We expected that our experimental plots of 25 × 12.5 are too small to measure differences between the flowering regimes for *I. alternans*. In contrast, *Trichogramma* species are minute and their active radius is probably more limited. At least with this group of egg parasitoids we would have expected differences between the different greencover types. The absence of an effect of greencover crops on *Trichogramma* suggests that the greencover zone is ecologically only poorly linked with the grape canopy for these parasiotids, a hypothesis which other authors already confirmed [39]. We found the species *T. pintoi* only in the surrounding vegetation but not in the vineyard. This species and *T. brassicae,* a species we only found within the vineyard, were so rarely detected that we did not consider them as key parasitoids. The plant protection regime within the vineyard apparently did not change the species composition in a way that one of the two key parasitoids *T. evanescence* and *T. cacoeciae* disappeared from the vineyard and stayed present in the untreated surroundings. Therefore, we think that there was no species loss due to plant protection products within the vineyards that might have altered the trial results. The same is true for *I. alternans*, a parasitoid detected in the surrounding vegetation as well as in the vineyards. It seems unlikely that our findings are the results of side effects of pesticides because that might have decreased parasitoid diversity and activity.

In contrast to these findings, only nonspecific predators showed differentiated activity in the different plots in 2014 (not considered in 2013). As predatory mite densities did not correspond with egg predation rates, they are unlikely to be important predators of *L. botrana* eggs. Buchholz & Schruft [40] found in feeding experiments that Chrysopidae, Forficulidae, Nabidae and Aranaeae are probably important egg predators in vineyards and also showed effects of different soil treatment on Forficulidae. Roltsch et al. [41] found that certain spider species move between greencover and the canopy of Californian vineyards. Exclusion of spiders from the grape canopy reduces predation rates on leafhoppers [42]. Thus, spiders may link the food web of the greencover and the vineyard canopy. However, it is unclear to what degree spiders contributed to the predation of *L. botrana* eggs in the current study. Unfortunately, the abundance of the mentioned predators could not be quantified in this study with the methods we applied. Further camera studies are necessary to find out the identity of the nonspecific predators that are enhanced by the greencover crops.

There was no clear effect of greencover crops on egg- and pupal parasitoids or on predatory mites over the two years. Only the predation of eggs changed in 2014 between sown and spontaneously growing vegetation. The parasitism of pupae and eggs fluctuated strongly and peaked in autumn 2014 for both pest stages. Still, there were no significant differences in the efficiency of the parasitoids in the different plots with the chosen greencover crops *Phacelia tanacetifolia* and *Fagopyrum esculentum*. The technique to use greencover crops to enhance natural enemies of *L. botrana* still needs further development in spite of the existence of such practices for over a century [7]. Catoni [43] found that the rate of parasitism of *L. botrana* is increased near coniferous trees. Schwangart [44] found that in traditional mixed cultures of Southern Tirol, parasitism is much higher than in vineyards of the Palatinate region in Germany were grape growers are obliged by law to keep their vineyards free of weeds. Schade [45] found that the presence of stinging nettle (*Urtica dioica*) in vineyards can lead to higher parasitism by the egg parasitoid *Trichogramma semblidis*. 

The strong differences between regions suggest that greencover crops for improved pest control need to be tailored to the regional situation with its specific pool of natural enemies and climatic conditions. Also, the landscape elements of the surrounding habitats might play a strong role in natural enemy activity [46,47]. Native plants can favor pest control by natural enemies more effectively than non-native greencover crops [48]. Thus, greencover crops composed of native plant species could be superior to the non-native *P. tanacetifolia* and *F. esculentum* used in this study. Another aspect is that predators in the greencover crop may not automatically switch to the grape canopy as long as there is enough prey in the greencover itself. This might open new approaches to future habitat management programs. Cutting greencovers can make insects change their stratum as it is already known for occasional vineyard pests like the vector of the bois noir disease *Hyalesthes obsoletus*. This insect only feeds on grapevine if their host plants are suppressed by herbicides [49]. In control efforts against the leafhopper *Erythroneura elegantula* [50], we found a deeper understanding of such switching mechanisms for predators and parasitoids from greencover crops to the grape canopy after mowing. Mowing the flowering greencover crop at a crucial moment might open new approaches to control *L. botrana* in viticulture by habitat management.

## 5. Conclusions

Greencover crops enhanced predation of *L. botrana* eggs, but parasitism was unaffected. As predatory mites did not show as strong an increase in greencover crops as egg predation, additional studies are necessary to identify the dominant predators of *L. botrana* eggs. Pest control benefits of greencover crops were minor compared to the strong within- and between-year variation in arthropod activity in the studied vineyard. Part of this variation could be related to the coincidence of greencover crop flowering and parasitoid activity between the two study years. The observed differences in predation between different greencover crop treatments might not be effective enough for the grower to keep the pest under the threshold level. In future studies, cost-benefit analyses are necessary to assert that planting greencover crops can be more sustainable than working with spontaneously grown vegetation. 

## Figures and Tables

**Table 1:** Overview on the *Trichogramma* species within the trial in the year 2014.

Species	Sum	2x	1x	Spontaneous	Surroundings
*T. evanescens*	888	90	67	65	666
*T. cacoeciae*	1712	296	101	420	895
*T. pintoi*	27				27
*T. brassicae*	1	1			
Not determined	181	31		6	144
Total number	2809	418	168	491	1732

## Supplementary Materials

The following are available online at www.mdpi.com/2075-4450/8/4/121/s1, Figure S1: Sampling Plan within greencover crop trial in Siebeldingen, Table S1: Active ingredients of the fungicides applied in 2013 and 2014, Table S2: Species composition of adult predatory mites in 2014. Table S3: Diversity of the leaf mesofauna in relation to the type of greencover in 2014.

## References

[1] Isaacs R., Tuell J., Fiedler A., Gardiner M., Landis D. (2009). Maximizing arthropod-mediated ecosystem services in agricultural landscapes: The role of native plants. Front. Ecol. Environ..

[2] Bommarco R., Kleijn D., Potts S.G. (2013). Ecological intensification: Harnessing ecosystem services for food security. Trends Ecol. Evol..

[3] Tschumi M., Albrecht M., Collatz J., Dubsky V., Entling M.H., Jacot K.A. (2016). Tailored flower strips promote natural enemy biodiversity and pest control in potato crops. J. Appl. Ecol..

[4] Irvin N.A., Bristline-East A., Hoddle M.S. (2016). The effect of an irrigated buckwheat cover crop on grape vine productivity, and beneficial insect and grape pest abundance in southern California. Biol. Control.

[5] Begum M., Gurr G.M., Wratten S.D., Hedberg P.R., Nicol H.I. (2006). Using selective food plants to maximize biological control of vineyard pests. J. Appl. Ecol..

[6] Gurr G.M., Wratten S.D., Landis D.A., You M. (2017). Habitat Management to Suppress Pest Populations: Progress and Prospects. Annu. Rev. Entomol..

[7] Silvestri P. (1912). Contribuzioni alla conoscenza degli insette dannosi et dei loro simbionti. IIIme, La Tignoletta dell’uva (*Polychrosis botrana* Schiff.) cun un cenno sulla Tignolla dell’uva (*Conchylis ambiguella* Hübn). Boll. Lab. Zool. Gen. Agrar. Portici.

[8] Hofmann U., Köpfer P., Werner A. (1995). Ökologischer Weinbau.

[9] Lorenzon M., Pozzebon A., Duso C. (2015). Feeding habits of overwintered predatory mites inhabiting European vineyards. BioControl.

[10] Baggen L.R., Gurr G.M. (1998). The influence of food on *Copidosoma koehleri* (Hymenoptera: Encyrtidae), and the use of flowering plants as a habitat management tool to enhance biological control of potato moth, *Phthorimaea operculella* (Lepidoptera: Gelechiidae). Biol. Control.

[11] Fiedler A.K., Landis D.A., Wratten S.D. (2008). Maximizing ecosystem services from conservation biological control: The role of habitat management. Biol. Control.

[12] Andow D.A. (1991). Vegetational diversity and arthropod population response. Annu. Rev. Entomol..

[13] Berndt L.A., Wratten S.D., Scarratt S.L. (2006). The influence of floral resource subsidies on parasitism rates of leafrolers (Lepidoptera: Tortricidae) in New Zealand vineyards. Biol. Control.

[14] Gerson U., Smiley R.L., Ochoa R. (2003). Mites (Acari) for Pest Control.

[15] Pertot I., Caffi T., Rossi V., Mugnai L., Hoffmann C., Grando M.S., Gary C., Lafond D., Duso C., Thiery D. (2017). A critical review of plant protection tools for reducing pesticide use on grapevine and new perspectives for the implementation of IPM in viticulture. Crop Prot..

[16] Tiso R., Butturini A. (1997). Possibilità di impiego di un modello fenologico per *Lobesia botrana* Schiff. nella difesa della vite. Atti dei Convegni XXII MOMEVI.

[17] Markheiser A., Rid M., Biancu S., Gross J., Hoffmann C. (2017). Physical factors influencing the oviposition behavior of European grapevine moths *Lobesia botrana* and *Eupoecilia ambiguella*. J. Appl. Entomol..

[18] Hoffmann C., Michl G. (2003). Parasitoide von Traubenwicklern—Ein Werkzeug der natürlichen Schädlingsregulation?. Deutsches Weinbau-Jahrbuch.

[19] Hoffmann C. (2008). Simulation of Lobesia-botrana-egg-laying for autecological and insecticide studies. IOBC-WPRS Bull..

[20] Gonzalez R. (2015). Lobesia botrana (D.&S.) y Otras Polllas Plagas de la vid en Chile (Lepidoptera: Tortricidae).

[21] Townes H. (1969). The Genera of Ichneumonidae, Part 1.

[22] Townes H. (1970). The Genera of Ichneumonidae, Part 2.

[23] Townes H. (1970). The Genera of Ichneumonidae, Part 3.

[24] Townes H. (1971). The Genera of Ichneumonidae, Part 4.

[25] Kolarov Y. (1997). Fauna Bulgarica: Pimplinae, Xoridinae, Acaeninae, Collyriinae.

[26] Sumer F., Tuncbilek A.S., Oztemiz S., Pintureau B., Rugman-Jones P.F., Stouthamer R. (2009). A molecular key to the common species of Trichogramma of the Mediterranean region. BioControl.

[27] Lucchi A., Scaramozzino P.L., Michl G., Loni A., Hoffmann C. (2016). The first record in Italy of *Trichogramma cordubense* Vargas & Cabello 1985 (Hymenoptera Trichogrammatidae) emerging from eggs of *Lobesia botrana* (Denis & Schiffermüller, 1775) (Lepidoptera Tortricidae). VITIS J. Grapevine Res..

[28] Stouthamer R., Hu J., van Kan F.J.P.M., Platner G.R., Pinto J.D. (1999). The utility of internally transcribed spacer 2 DNA sequences of the nuclear ribosomal gene for distinguishing sibling species of *Trichogramma*. BioControl.

[29] Fusu L., Mitroiu M.D. (2016). Fauna Europaea: Trichogrammatidae. Fauna Europaea: Chalcidoidea.

[30] Hill G.K., Schlamp H.A. (1984). Der Einsatz der Waschmethode zur Ermittlung des Raubmilbenbesatzes auf Rebblättern. Weinwissenschaften.

[31] Krantz G.W. (1978). A Manual of Acarology.

[32] Karg W. (1997). Stammesentwicklung und Lebensweise von Raubmilben. Mikrokosmos.

[33] R Core Team (2015). R: A Language and Environment for Statistical Computing.

[34] Lorenzon M., Pozzebon A., Duso C. (2012). Effects of potential food sources on biological and demographic parameters of the predatory mites *Kampimodromus aberrans*, *Typhlodromus pyri* and *Amblyseius andersoni*. Exp. Appl. Acarol..

[35] Karban R., English-Loeb G., Walker M.A., Thaler J. (1995). Abundance of phytoseiid mites on *Vitis* species: Effects of leaf hairs, domatia, prey abundance and plant phylogeny. Exp. Appl. Acarol..

[36] Kreiter S., Tixier M.-S., Croft B.A., Auger P., Barret D. (2002). Plants and leaf characteristics influencing the predaceous mite *Kampimodromus aberrans* (Acari: Phytoseiidae) in habitats surrounding vineyards. Environ. Entomol..

[37] Zhang G.R., Zimmermann O., Hassan S.A. (2004). Pollen as a source of food for egg parasitoids of the genus *Trichogramma* (Hymenoptera: Trichogrammatidae). Biocontrol Sci. Technol..

[38] Araújo E.D., Costa M., Chaud-Netto J., Fowler H.G. (2004). Body size and flight distance in stingless bees (Hymenoptera: Meliponini): Inference of flight range and possible ecological implications. Braz. J. Biol..

[39] Wilson H., Miles A.F., Daane K.M., Altieri M.A. (2017). Landscape diversity and crop vigor outweigh influence of local diversification on biological control of a vineyard pest. Ecosphere.

[40] Buchholz U., Schruft G. (1994). Räuberische Arthropoden auf Blüten und Früchten der Weinrebe (*Vitis vinifera* L.) als Antagonisten des Einbindigen Traubenwicklers (*Eupoecilia ambiguella* Hbn.) (Lep., Cochylidae). J. Appl. Entomol..

[41] Roltsch W.R., Hanna F., Zalom H., Shorey H., Mayse M., Pickett C., Bugg R. (1998). Spiders and vineyard habitat relationship in central California. Enhancing Natural Control of Arthropods through Habitat Management.

[42] Costello M.J., Daane K. (2003). Spider and Leafhopper (*Erythroneura* spp.) Response to Vineyard Ground Cover. Environ. Entomol..

[43] Catoni G. (1914). Die Traubenwickler (*Polychrosis botrana* Schiff. und *Conchylis ambiguella* Hübn.) und ihre natürlichen Feinde in Südtyrol. Z. Angew. Entomol..

[44] Schwangart F. (1913). Über die Traubenwickler und ihre Bekämpfung, mit Berücksichtigung Natürlicher Bekämpfungsfaktoren.

[45] Schade M. (1990). Untersuchungen zur Förderung des einheimischen Eiparasitoiden *Trichogramma semblidis* (Auriv.) (Hym. Trichogrammatidae) als Natürlicher Feind Beider Traubenwicklerarten im Ahrtal. Ph.D. Thesis.

[46] Schmidt M.H., Thies C., Nentwig W., Tscharntke T. (2008). Contrasting responses of arable spiders to the landscape matrix at different spatial scales. J. Biogeogr..

[47] Chaplin-Kramer R., O’Rourke M.E., Blitzer E.J., Kremen C. (2011). A meta-analysis of crop pest and natural enemy response to landscape complexity. Ecol. Lett..

[48] Danne A., Thomson L.J., Sharely D.J., Penfold C.M., Hoffmann A.A. (2010). Effects of Native Grass Cover Crops on Beneficial and Pest Invertebrates in Australian Vineyards. Environ. Entomol..

[49] Maixner M. (2005). Biology of *Hyalesthes obsoletus* and approaches to control this soilborne vector of Bois noir disease. IOBC-WPRS Bull..

[50] Nicholls C.I., Parella M.P., Altieri M.A. (2000). Reducing the abundance of leafhoppers and thrips in a northern California organic vineyard through maintenance of full season floral diversity with summer cover crops. Agric. For. Entomol..

