# Supplementary material for: Can Flowering Greencover Crops Promote Biological Control in German Vineyards?
Source: Insects. 2017 Nov 3;8(4):121. doi: 10.3390/insects8040121 (PMC5746804; doi:10.3390/insects8040121)
Supplement: Supplementary file 1 [file insects-08-00121-s001.pdf]

insects

Can Flowering Greencover Crops Promote Biological Control in German Vineyards?

Christoph Hoffmann [1,*], Janine Köckerling [1], Sandra Biancu [1], Thomas Gramm [1], Gertraud Michl [1] and Martin H. Entling [2]

Supplementary Materials:

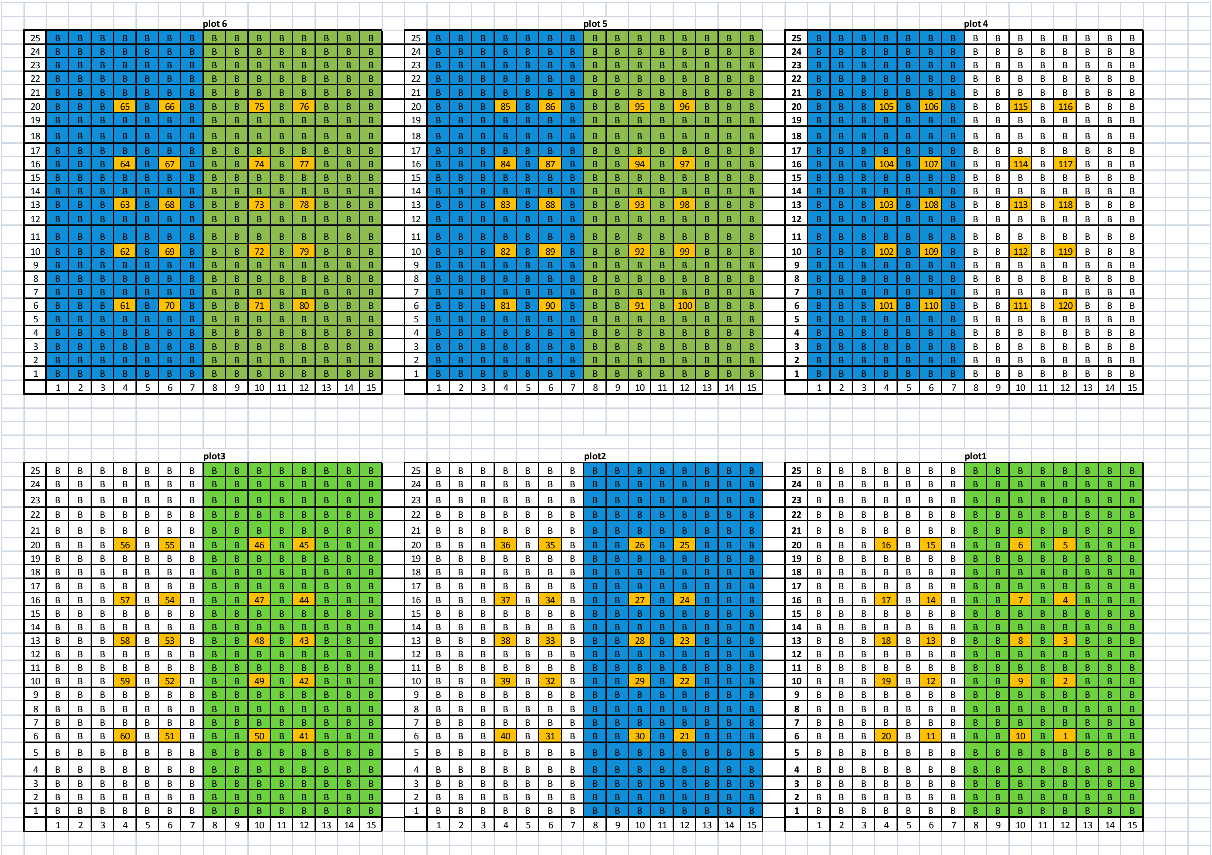

Figure S1. Sampling Plan within greencover trial in Siebeldingen. White: Spontaneously growing vegetation; Blue: 1x -; Green: 2x sowing of *Phacelia* & *Fagopyrum* The yellow rectangles are the positions where the cages and egg stripes were exposed weekly between 07.06. -15.10.2013 and between 26.03.-16.09.2014.

Table S1. Active ingredients of the fungicides applied in 2013 and 2014.

2013	2014
ametoctradin; boscalid; cyprodinil; dimethomorph; fludioxonil; folpet; kresoxim-methyl; metiram; metrafenone; myclobutanil; sulphur	ametoctradin; cyflufenamid; difenoconazole; dimethomorph; dithianon; fluopyram; folpet; metiram; metrafenone; myclobutanil; proquinazid; sulphur; tebuconazole

Table S2. Species composition of adult predatory mites in 2014 (Randomly sampled from the total catch).

**Month 2014**	**Phytoseiidae**			**Tydeidae**
	Typhlodromus pyri	*Paraseiulus soleiger*	*Euseiulus finlandicus*	*Tydeus goetzi*
June	123	8	0	29
July	119	1	0	23
August	159	3	0	20
September	178	2	2	33
October	158	6	3	57
Sum	737	20	5	162

Table S3: Diversity of the leaf mesofauna in relation to the type of greencover in 2014. No major changes in the leaf mesofauna appear between the control and the different sowings of greencover crops *Fagopyrum esculentum* and *Phacelia tanacetifolia*.

**Month** / **Treatment**		***Calepitrimerus vitis***	***Colomerus vitis***	**Phytoseiidae**	**Tydeidae**	**Thysanoptera**	***Empoasca vitis***	***P. corni***	**Collembola**
**June**									
2 drillings	∑	**98**	**43**	**2683**	**294**	**27**	**24**	**9**	**0**
1 drilling	∑	**119**	**35**	**2612**	**1041**	**38**	**30**	**12**	**1**
spontaneously grown veg.	∑	**192**	**80**	**2387**	**1417**	**54**	**30**	**34**	**5**
**July**									
2 drillings	∑	**37**	**14**	**1189**	**112**	**4**	**46**	**4**	**6**
1 drilling	∑	**28**	**7**	**1304**	**164**	**7**	**38**	**6**	**4**
spontaneously grown veg.	∑	**31**	**9**	**1202**	**166**	**3**	**43**	**5**	**5**
**August**									
2 drillings	∑	**8**	**6**	**1131**	**68**	**1**	**9**	**3**	**2**
1 drilling	∑	**14**	**5**	**1120**	**101**	**2**	**7**	**3**	**2**
spontaneously grown veg.	∑	**19**	**5**	**993**	**109**	**3**	**19**	**3**	**3**
**September**									
2 drillings	∑	**11**	**9**	**1242**	**218**	**1**	**6**	**0**	**0**
1 drilling	∑	**6**	**2**	**1181**	**252**	**2**	**7**	**1**	**2**
spontaneously grown veg.	∑	**9**	**5**	**965**	**342**	**2**	**3**	**1**	**2**
**October**									
2 drillings	∑	**1**	**0**	**556**	**760**	**1**	**0**	**0**	**0**
1 drilling	∑	**1**	**0**	**554**	**892**	**3**	**0**	**0**	**4**
spontaneously grown veg.	∑	**1**	**1**	**417**	**700**	**7**	**0**	**1**	**0**

© 2017 by the authors. Submitted for possible open access publication under the terms and conditions of the Creative Commons Attribution (CC BY) license (http://creativecommons.org/licenses/by/4.0/).
